# Re-defining professionalism in medicine in an era of rapid change: a modified Delphi study

**DOI:** 10.3389/fmed.2025.1686745

**Published:** 2026-01-20

**Authors:** Amy M. Sullivan, Ling Hsiao, Richard M. Schwartzstein, Margaret (Molly) M. Hayes, Cullen D. Jackson, Daniele D. Ölveczky, Daniel N. Ricotta, Carrie Tibbles, K. Meredith Atkins

**Affiliations:** 1 Department of Medicine, Beth Israel Deaconess Medical Center and Harvard Medical School, Boston, MA, United States; 2 Shapiro Institute for Education and Research, Beth Israel Deaconess Medical Center, Boston, MA, United States; 3 Department of Anaesthesia, Beth Israel Deaconess Medical Center and Harvard Medical School, Boston, MA, United States; 4 Department of Emergency Medicine, Beth Israel Deaconess Medical Center and Harvard Medical School, Boston, MA, United States; 5 Department of Obstetrics, Gynecology and Reproductive Biology, Beth Israel Deaconess Medical Center and Harvard Medical School, Boston, MA, United States

**Keywords:** professionalism, modified Delphi approach, undergraduate medical education, graduate medical education, faculty development

## Abstract

**Introduction:**

Medical professionalism has traditionally been defined by core standards for practitioners, yet consensus on its defining elements remains limited. Shifts in society, medical practice, and trainee perspectives have influenced how professionalism is understood and applied. This study aimed to establish a contemporary, consensus-based framework for medical educators and learners.

**Methods:**

Using a modified Delphi approach, 39 medical education experts from eight U.S. medical schools participated in three survey rounds and one in-person session. Participants rated 51 behaviorally-based items categorized under four domains: commitments to patients, colleagues, institutions/society, and self. Items were rated “essential,” “important but not essential,” or “not important,” with consensus defined as 70% agreement.

**Results:**

Consensus was reached on 24 “essential” elements emphasizing patient-centered care, ethical practice, equitable care, communication, and cultural humility. Participants highlighted the importance of a shared framework while recognizing the risks of bias and the need for contextual sensitivity. Items related to physician self-sacrifice, attire, and social justice failed to reach consensus, reflecting concerns about burnout, subjectivity, and scope. Emphasis was placed on fostering professionalism through dialog, reflection, and context-aware evaluation.

**Conclusion:**

The resulting framework captures evolving perspectives on professionalism, offering educators practical, adaptable guidance for teaching, reflection, and assessment across diverse educational settings.

## Introduction

For centuries, medicine has been a profession governed by a code of conduct that has been taught and reinforced through role modeling, mentoring, and formal curricula (1–3). While many elements of medical professionalism have endured for centuries, the changing nature of society, medical practice, and the relationship between physicians and patients have particularly affected how professionalism is conceptualized and practiced in recent years; increasing focus in the U.S. on issues related to race, diversity, mental health, and healthcare disparities has highlighted vulnerabilities and inequities in the healthcare system (4). The COVID-19 pandemic further exposed these challenges, underscoring the need for physicians to address issues of their own wellbeing and the need to provide equitable care with limited resources (5). Advances in technology have brought new opportunities and challenges for practice, while the nearly ubiquitous use of social media has created a new arena for discussion and debate about professional and ethical practice in both scholarly and public discourse (6, 7). These multiple forces of social and cultural change have resulted in increasing friction between students/trainees and faculty, necessitating efforts to revise, expand, and clarify the evolving construct of professionalism.

For educators working to teach and provide feedback on professional values and behaviors for the next generation of clinicians, both consensus and a common language are needed to guide curricula that respond to these contemporary issues. While there may be agreement about many core principles, an updated consensus definition of professionalism that reflects current challenges and realities in U.S. medical education is lacking (1–3, 8–13). Further, some assessments of professionalism have drawn criticism for failing to account for learners’ diverse backgrounds and for focusing on deviation from norms that may seem poorly defined, comprising part of the hidden curriculum (13), or at variance with the learners’ values (11). Therefore, to minimize conflict among faculty, students, and residents over these issues and the associated risks of harm to the learning environment and patient care, educators need guidance on ways in which professional norms can be adapted to today’s learners.

It is within this context that the Shapiro Institute for Education and Research at Harvard Medical School and Beth Israel Deaconess Medical Center, with support from the Association of American Medical Schools, convened teams from eight medical schools across the United States as part of the 3 day Millennium Conference Series (14) to discuss professionalism and professional identity formation during a period of pandemics and social unrest. Due to the complexity of this topic, we chose, as a prelude to the Conference, to develop a characterization of the essential elements of professionalism relevant to our present times via a consensus-driven process. By foregrounding the social and cultural dimensions of how professionalism is understood and practiced, we aimed to generate a deeply considered, nuanced perspective of professionalism that would support educators and learners and explicitly address the changes and challenges of contemporary society. Our goal was to define active educators’ perspectives of critical components of professionalism applicable *now* to the education of *all* physicians in training, whether they are academically oriented, committed to clinical practice, or focused on advocacy and public policy.

## Methods

We conducted a modified Delphi study to develop a consensus approach to professionalism that would serve the needs of educators across the continuum of medical education. Delphi methodologies typically include iterative rounds of data collection and feedback with an identified group of experts and a predefined threshold (percent agreement, here set at 70%) to determine consensus (15). We modified the Delphi by (a) developing a list of items before the first round of data collection based on a literature review, and (b) conducting extended face-to-face discussions of items as part of Round 3 of the study. This latter modification has been used in cases where phenomena under study can be better understood through extended discussions about different perspectives, controversies, and contexts that would not be captured in surveys (16).

Our methodology was informed by the philosophical paradigm of constructivism (17) and the theoretical framework of Communities of Practice (18). Constructivism aligns with our understanding of professionalism as a socially constructed concept that evolves through the development of a shared understanding among expert educators. Communities of Practice informed our in-person discussions in Round 3, in which participants explored and deepened their conceptualization of professionalism through extended discussions of experiences and challenges in teaching and assessing professionalism, which framed our in-person small- and large-group discussions. We used the CreDeS Delphi reporting guidelines in the design and reporting of this study (19). Our study team comprised leaders and experts in medical education and research, all of whom were actively involved in the teaching and evaluation of professionalism in undergraduate (UME) and/or graduate (GME) medical education. The study received a determination of “not human subjects research” by the BIDMC Committee on Clinical Investigations.

### Item generation

We began our study with a literature review, focusing on existing definitions of professionalism published within the past 10 years; in addition, we included the Physician Charter of the American Board of Internal Medicine (20) and other seminal studies (3, 10, 21, 22), which predated this time interval. We used PubMed, ERIC, PsycINFO, and Google Scholar as our primary search platforms. The research team (AMS, LH, KMA, MMH, CT, DR, and RMS) created an annotated bibliography of all identified articles and met biweekly for 6 months to determine which elements to include in the surveys. In our review and ongoing discussions, we paid particular attention to the inclusion of elements that addressed potential societal influences on changing views of professionalism. For example, rising attention to equity and health disparities shaped items on cultural humility and provision of equitable care; widespread concerns about physician burnout motivated items addressing physical and mental wellbeing; and the growing influence of social media informed items concerning responsible use of technology.

In our review, we found overlap as well as clear differences in approaches to professionalism. Existing constructs ranged from overarching principles and somewhat abstract, individual-level values, to behaviorally oriented principles and commitments that, in theory, would lend themselves more easily to use in curriculum development and learner assessment (10, 23). We maintained a running list of (1) items that were mentioned repeatedly in some form across multiple studies, (2) items generated from large-scale efforts to define professionalism, (3) scales and/or items described by highly-cited authors in the field, and (4) items emerging from ongoing discussions among our research team (search terms and generated list in Supplementary Digital Appendix 1). We then compiled a full list of potential items, deleting duplicates and rewording for consistency. We chose four overarching categories—commitments to patients, colleagues, institution and society, and self—both to reduce cognitive burden for our participants and to align with similar groupings suggested by CanMEDS, ABIM, and other organizations. While the initial items were grouped in four categories as described above, we did not impose a framework or theoretical construct on the process; rather, we preferred to see if a framework emerged from the consensus process.

### Survey development and testing

Our survey development process aimed to maximize comprehensiveness, relevance, and clarity while minimizing redundancy and cognitive load for respondents (24). We assessed each item for conceptual clarity and appropriate level of specificity, complexity, and length. We included 51 items in the initial survey, sorted into four categories: commitments to (1) patients (18 items); (2) colleagues (17 items); (3) institution and society (9 items); and (4) self (7 items). Items were phrased in behavioral terms to create elements of professionalism that could be taught, supported, assessed, and developed over time (Supplementary Digital Appendix 2).

Survey respondents were instructed to identify “essential” dimensions of professionalism that could guide the development of curricula to teach and assess professionalism. For each item, we used a 3-point response scale with choices of “essential,” “important,” or “not important” for professionalism. Respondents were also asked to propose new items, combine items, or suggest rewordings. We conducted cognitive interviews with colleagues who represented the range of participants in the study but were not part of the Millennium Conference, and revised it as needed (25).

### Selection of experts

Participation in one of the eight school teams selected for the 2023 Millennium Conference (MC) was the single-study inclusion criterion. The MC selection process was based on both school- and team-level criteria. At the school level, applications were rated on documented school-level commitment to supporting initiatives in professionalism and professional identity formation. At the team level, we sought to include multi-professional teams of educational leaders who represented UME and GME and members with experience and expertise in teaching and evaluating professionalism and/or professional identity formation. Student and resident representatives were welcome, but not required, to be included in the application. In consultation with representatives from the AAMC leadership, we sought to include a range of North American medical schools representing both public and private institutions, varied geographic regions, and maximizing racial and ethnic diversity at the student and resident levels (one team represented a historically black university). Of the 23 schools that applied to attend the conference, 7 schools with 4–5 team members each were selected for participation; in addition, a team from Harvard Medical School (HMS) was included, given the link between the Shapiro Institute and HMS. The schools represented northern, southern, eastern, and midwestern regions of the United States as well as both public and private schools. Table 1 shows the demographics of the participants; we did not ask participants for self-identified race.

**Table 1:** Academic rank, education role, and professional training of expert panelists from eight U.S. medical schools, Professionalism Delphi Study, Shapiro Institute of Education and Research, 2023 (*n* = 39).

Variables	*n* = 39	*n* (%)
Gender		
Female	27 (69.2)	
Male	12 (30.7)	
Academic rank		
Clinical Associate Professor	3	(8)
Assistant Professor	5	(13)
Associate Professor	16	(41)
Professor	13	(33)
Education role		
Program/Associate Program Director	11	(28)
Course/Clerkship/Curriculum Director	6	(15)
Fellowship Director	3	(8)
Associate Dean (UME/GME/Student Affairs/Assessment)	15	(39)
Vice Chair of Education	1	(3)
PhD/MD students	2	(5)
Professional training		
MD	26	(67)
Masters of Public Health	4	(10)
Master of Science/Masters of Medical Sciences	4	(10)
Master of Health Professions Education	2	(5)
PhD	4	(10)

## Data collection

### Rounds 1 and 2: online data collection and participant feedback

We defined “consensus” in each round as 70% agreement in any response category (“essential,” “important,” or “not important”) (15, 26). For the first two rounds, we sent out the anonymous Delphi survey to all participants using the online Qualtrics® survey platform (Provo, UT). We summarized results after each round, removing items that reached our pre-determined threshold of agreement. Round 2 included a report of items that reached consensus, a summary of open-ended item responses, and a shortened survey that included items that had not reached consensus (data report also in Supplementary Digital Appendix 2).

### Round 3: in-person discussions and final survey

The third round involved an in-person meeting with participants at an off-site conference center on 2 May 2023. It was designed to foster face-to-face discussion of Round 2 results, review items that did not reach consensus, and discuss controversies or concerns about the overall results. Plans for Round 3 included small- and large-group facilitated discussions and a final survey to adjudicate remaining items.

For the breakout group meetings, we created a data collection form for participants to review items that had not reached consensus, as well as open-ended fields for participants to suggest rewording, new items, or share comments about the content or meaning of existing items (Supplementary Digital Appendix 3). Six members of our research group (AMS, KMA, CJ, CT, MMH, and DO) served as group facilitators. Facilitators met before the start of the conference to review the purpose and conduct of these meetings. One member (RMS) facilitated a large group (comprising all participants) discussion following the breakout sessions.

### Data analysis

We calculated response rates and percent agreement for closed-ended items in each round of data collection. For qualitative data, LH and AMS transcribed data and carried out (1) a content analysis for open-ended survey items (27) and (2) thematic analysis of data from small- and large-group in-person discussions (such as small-group facilitator’s notes and large group discussion notes) (28). Our survey content analysis focused on categorizing participant comments and documenting frequencies of comments from highest-to-lowest frequency of occurrence.

For qualitative analysis of the in-person discussions, we used the Framework Approach for thematic analysis (27), which allows for both deductive (e.g., *a priori* themes from the survey comments) and inductive (drawn directly from discussion data) approaches to analysis. Steps in this approach include: familiarization with the data, where researchers immerse themselves in the data by reading transcripts and meeting notes; identification of an initial set of themes; creation of a codebook to systematically code the data; and looking for recurring patterns and connections among identified themes. Two non-clinician investigators with expertise in qualitative research (AS and LH) met regularly to identify and discuss themes. We carried out this analysis over a series of meetings and noted whether identified themes aligned with the survey comments or raised new issues. We addressed the trustworthiness of findings through ongoing discussion with the larger study team, all of whom were present at the conference, and feedback to the Delphi panel for comments (29). To minimize potential bias, the qualitative analysts and overall study team employed ongoing practices of reflexivity (ongoing attention to and discussion of how our own experiences and biases might influence our inferences) in both analytic memos and study team discussions (30).

## Results

All 39 conference participants from the eight schools were invited to serve as expert panelists. Rounds 1 and 2 were completed by 35 (89.7% response rate) and 34 respondents (87.2%), respectively. Three individuals from the initial panel withdrew from conference participation after Round 2 due to scheduling conflicts, and one new participant was added. Of the 37 participants who attended the conference, 30 (81.1%) completed the survey in Round 3. Data were collected between March and May 2023.

The 39 conference participants were experienced educators leading initiatives at their medical schools and GME programs that focused on professionalism and professional identity formation (Table 1). Thirty-nine percent (15/39) were associate deans in undergraduate, graduate medical education, or student affairs, 28% (11/39) served as program directors, and 15% (6/39) were course/clerkship directors. The majority (67%, 26/39) were clinician educators, and 10% (4/39) were Ph.D. educators. Many taught courses and published research centered on professionalism, such as promoting student reflections about professional identity. Two participants were senior students distinguished in their community outreach or research activities on student professionalism.

### Rounds 1 and 2

Table 2 shows the results for each survey round. In Round 1, 19/51 items reached consensus, with 17 rated as “essential” and 2 as “important.” Based on category, nearly two-thirds (11/18) of the items in Commitments to Patients and 3/6 items in Commitments to Self-reached consensus. A minority of items in the Commitments to Colleagues (3/16) and Commitments to Institution and Society (2/11) reached consensus in this round. The most common reasons noted for items that did not reach consensus in Round 1 were lack of context or redundancy with other items (see Table 3). An additional four items reached consensus in Round 2, with one of these rated as “essential” and three as “important”; two of these were in the Patient category, and one each was in the Colleagues and Institution categories.

**Table 2:** Consensus decisions and summary of the Professionalism Delphi Survey Rounds 1–3, Shapiro Institute of Education and Research, 2023.

Item#	Element	Consensus decisions (% agreement)		
		Round 1 decisions (Total: 35/39 experts)	Round 2 decisions (Total: 34/39 experts)	Round 3 decisions (Total: 30/37 experts)
Commitments to patients				
P1	Speak honestly with patients.	Essential (97.1%)	–	–
P2	Demonstrate respect for patient confidentiality.	Essential (94.3%)	–	–
P3	Strive for competency and clinical excellence.	Essential (88.6%)	–	–
P4	Listen and respond to patients’ concerns.	Essential (88.6%)	–	–
P5	Minimize risks to patients.	Essential (85.7%)	–	–
P6	Demonstrate respect for patient autonomy.	Essential (85.7%)	–	–
P7	Communicate clearly to patients.	Essential (85.7%)	–	–
P8	Convey compassion to patients.	Essential (80.0%)	–	–
P9	Establish appropriate boundaries in relationships with patients.	Essential (80.0%)	–	–
P10	Engage in mutual decision-making.	Essential (77.1%)	–	–
P11	Demonstrate cultural humility.	Original wording: “Demonstrate cultural proficiency.” NC = No consensus	Revised: “Demonstrate cultural humility.” Essential (82.4%)	–
P12	Attend to patient’s family needs.	Important (82.4%)	–	–
P13	Using technology, including social media, appropriately.	NC	Important (85.3%)	–
P14	Demonstrate respect for the values and identities of patients. Be curious and engaged with patients.	–	Suggested new item: “Demonstrate an attitude of curiosity and engagement with patients.” NC	Revised wording: “Demonstrate respect for the values and identities of patients. Be curious and engaged with patients.” Essential (83.3%)
P15	Manage conflicts of interests.	NC	NC	NC
P16	Be timely in completing medical records.	NC	NC	NC
P17	Prioritize patient care over physician’s own self-interests.	Original wording: “Prioritize patient care over one’s own needs.” NC	Revised wording: “Prioritize patient care over physician’s own self-interests.” NC	NC
P18	Demonstrate tolerance for ambiguity and uncertainty.	–	Suggested new item: “Demonstrate tolerance for ambiguity and uncertainty.” NC	NC
Commitments to colleagues				
C1	Commit to lifelong learning.	Essential (80.0%)	–	–
C2	Respond to feedback appropriately.	Essential (77.1%)	–	–
C3	Be conscientious.	Essential (74.3%)	–	–
C4	Dress appropriately.	NC	Important (75.8%)	–
C5	Be punctual.	NC	NC	Removed**
C6	Commit to staying current in scientific knowledge.	NC	NC	Removed
C7	Demonstrate respect for other specialties.	NC	NC	Removed
C8	Demonstrate respect for other clinical professions.	NC	NC	Removed
C9	Be a role model for trainees.	NC	NC	Removed
C10	Address evidence of unprofessional behavior.	Original wording: “Address evidence of unprofessional behavior.” NC	Revised wording: “Address evidence of unprofessional behavior appropriately.” NC	Removed
C11	Provide support to colleagues’ intellectual, emotional, and physical wellbeing.	Original wording: “Offer help to colleagues who may be struggling.” NC	Revised wording: “Provide support to colleagues’ intellectual, emotional, and physical wellbeing.” NC	Removed
C12	Use appropriate language.	NC	NC	Removed
C13	Request appropriate supervision.	NC	NC	Removed
C14	Be humble.	NC	NC	Removed
C15	Be responsive to colleagues’ needs.	NC	Removed in Round 2. Replaced by C11.	–
C16	Practice situational awareness	NC	NC	Removed
Commitments to institutions and society				
IS1	Act in accordance with a code of ethics.	Essential (85.7%)	–	–
IS2	Maintain competence.	Essential (82.9%)	–	–
IS3	Commit to practice that provides equitable care to all segments of the population.	–	Suggested new item: “Commit to practice that provides equal care to all segments of the population.” Suggestion to change ‘equal’ to “equitable care” instead.	Essential (73.3%)
IS4	Maximize access to care.	NC	Important (70.6%)	–
IS5	Optimize the quality of care even when access to needed clinical resources is constrained.	Original wording: “Manage limited resources for optimal patient outcomes.” NC	Revised wording: “Optimize the quality of care even when access to needed clinical resources is constrained.” NC	Removed. Replaced by IS4.
IS6	Promote social justice.	NC	NC	Removed. Replaced by IS3.
IS7	Be compliant with regulatory standards.	NC	NC	Removed
IS8	Debrief about error.	NC	NC	Removed
IS9	Commit to ongoing quality improvement.	NC	NC	Removed
IS10	Commit to training and teaching the next generation.	–	Suggested new item: “Commit to training and teaching the next generation.” NC	Removed
IS11	Be transparent.	NC	NC	Removed
Commitments to self				
S1	Address gaps in knowledge.	Essential (80.0%)	–	–
S2	Practice self-regulation.	Essential (80.0%)	–	–
S3	Practice self-reflection and commit to addressing your own biases.	Original wording: “Practice self-reflection.” NC	Revised wording: “Practice self-reflection, query your own biases, and be open to addressing them” NC	Revised wording: “Practice self-reflection and commit to addressing your own biases.” Essential (79.3%)
S4	Seek mentorship.	Important (77.1%)	–	–
S5	Maintain physical and mental wellbeing in order to care for your patients.	Original wording: “Practice self-care.” NC	Revised wording: “Maintain physical and mental wellbeing in order to care for your patients.” NC	NC
S6	Cultivate emotional intelligence.	NC	NC	Removed
Universal elements*				
U1	Act with integrity.	Essential (94.3%) in Commitments to Colleagues		Essential (96.7%)
U2	Demonstrate respect.	Essential (≥85%) across items in Commitments to Patients		Essential (85.0%)
U3	Be accountable.	Essential ≥82% across items in Commitments to Colleagues, Institutions & Society, and Self		Essential (82.0%)
U4	Act with humility.	–		NC
U5	Demonstrate curiosity.	–		NC

The original 39 participants, 3 withdrew from the study and conference. Reasons for non-response of other participants are unknown because surveys were anonymous. *These items reached consensus in Round 1 in one or more categories, and in Round 3 participants suggested that they be moved to a new, “universal” category that cut across all categories. **Items far from consensus in Round 2 were not included in the Round 3 survey. NC = No consensus; Light grey shading = Reached consensus as “essential”; Dark grey shading = Reached consensus as “important.”

**Table 3:** Elements that reached consensus as “essential” (≥70% benchmark) after Round 3 of the Professionalism Delphi Survey, Shapiro Institute of Education and Research, 2023.

Domain (*n* elements)	Essential elements
Universal elements (3)	Act with integrity. Demonstrate respect. Be accountable.
Commitments to patients (12)	Speak honestly with patients. Respect confidentiality. Strive for competence and clinical excellence. Listen and respond to concerns. Minimize risks. Respect autonomy. Communicate clearly. Convey compassion. Maintain appropriate boundaries. Engage in shared decision-making. Demonstrate cultural humility. Respect patients’ values and identities; be curious and engaged.
Commitments to colleagues (3)	Commit to lifelong learning. Respond appropriately to feedback. Be conscientious.
Commitments to institution and society (3)	Act in accordance with a code of ethics. Maintain competence. Provide equitable care to all populations.
Commitments to self (3)	Address knowledge gaps. Practice self-regulation. Reflect on and address personal biases.

### Round 3

#### Breakout group voting and discussions

During conference discussions, panelists in six breakout groups focused on the remaining 28 elements that had not reached consensus after Round 2. After breakout discussions, 24/28 elements remained far from consensus; these were subsequently removed from the item pool. Three items were identified as potential candidates for consensus as “essential” with rewording, and an additional five were suggested for consideration as “universal” elements, a newly formed category that cut across all survey categories. Proposed universal elements were: “Act with integrity”; “Act with humility”; “Demonstrate respect”; “Be accountable”; and “Demonstrate curiosity.” As one participant noted, the behaviorally worded items represented “an external manifestation of an inner grace,” and this called for an explicit articulation of the essential values that underlay the multiple behaviors.

### Large group discussion—thematic analysis

We identified three overarching themes in the 90-min large-group discussion that followed the breakout groups. The discussion was animated, included both agreement and disagreements, and participants from each of the schools participated fully in the discussion.

#### Theme 1: there are important tensions inherent in defining professionalism

In the large group discussion, participants expressed both resistance to and support for defining professionalism. Sentiments reflecting resistance to defining professionalism raised objections to the potential rigidity of definitions, punitive effect on learners from being identified as “unprofessional,” potential for paternalism and bias in faculty expectations and assumptions, and perceived tendency to dichotomize and reduce the construct into categories of “professional” and “unprofessional” behavior, most often with an emphasis on the latter. Elements of protectiveness were also present when participants spoke of their relationships with students and residents, e.g., “we love our learners,” and expressed concern for learner wellbeing if definitions were used to label them as unprofessional. Supportive statements for a consensus definition emphasized the need to teach and assess these attitudes and behaviors, and to have a shared lexicon or mental model among faculty and learners, and make explicit and cultivate positive attitudes and behaviors related to professionalism.

#### Theme 2: definitions of professionalism should be interpreted, discussed, and reflected upon in specific contexts

Participants were unified in the opinion that definitions should not be rigid or prescriptive; they also reiterated sentiments noted in the survey that considering context was essential in teaching, discussing, and assessing professionalism. Participants rejected items they felt were too specific, not allowing room for context or flexible interpretation, as well as items that were too abstract and subject to misinterpretation. Participants suggested using multiple examples to illustrate professionalism in discussions with learners, particularly with students who have limited clinical experience, to understand the variety, complexity, and contextual nature of professional behaviors in the workplace. Participants also emphasized the need for faculty and learner discussion and reflection to bring clarity to the construct.

#### Theme 3: faculty should emphasize the positive elements of professionalism in teaching, role modeling, and giving feedback to learners

Panelists recommended that faculty emphasize positive instances of professionalism and not assume students were “coming to medical school as fully formed professionals.” They suggested that faculty convey that conceptions of professionalism are meant to “emphasize the need to behave in a way that engenders trust and respect with patients and among team members.”

In discussing feedback about professionalism, participants also emphasized the need to give positive feedback to students who demonstrate professional behaviors and to recognize positive examples of professionalism in the classroom or workplace. In cases where constructive feedback was warranted, participants recommended that feedback about specific behaviors occur in the context of a discussion; for example, they encouraged faculty to ask students *why* they may have behaved in a particular way (such as being late for class or clinic) rather than make assumptions about a particular behavior as unprofessional.

#### Round 3 survey

**Figure 1:**
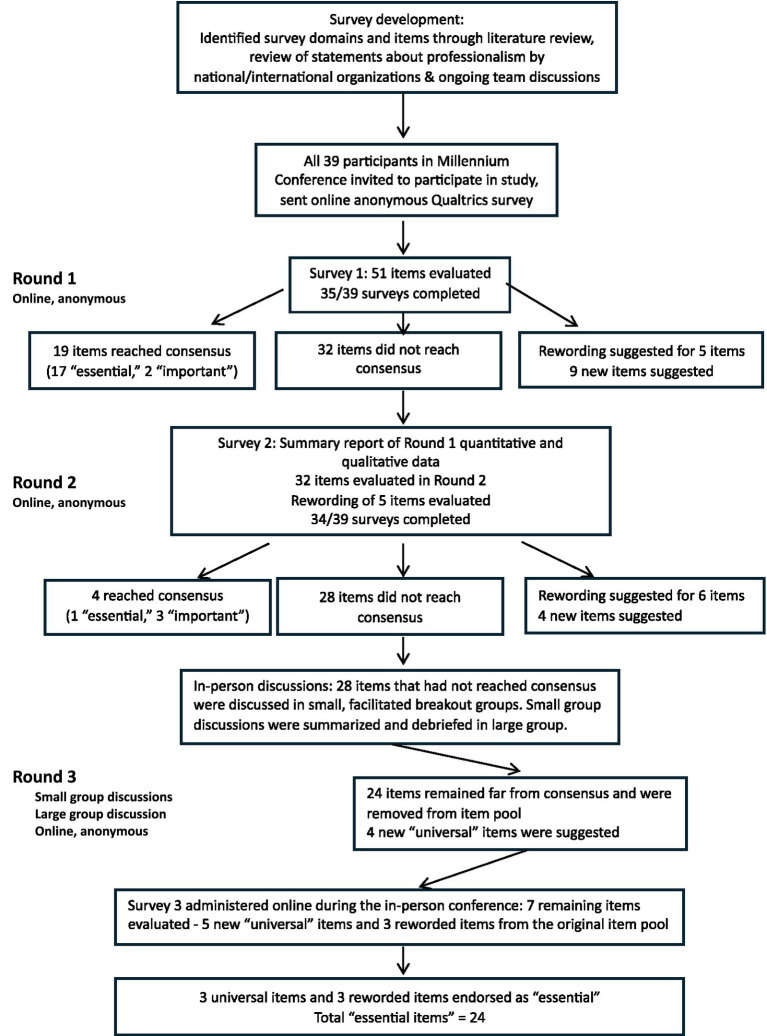
Item flow and decision logic for Professionalism Delphi Study, Shapiro Institute of Education and Research, 2023.

Immediately following the small- and large-group discussions, we compiled results and created and distributed a final survey that included new universal items and three close-to-consensus items needing rewording (Supplementary Digital Appendix 3). Panelists were asked which alternative wording they preferred and how they would rate the reworded item. The reworded elements reached consensus on the survey as essential (Table 2). The three universal elements that reached consensus as essential values were those addressing respect, integrity, and accountability. Curiosity and humility were considered too vague and open to multiple interpretations, and some participants thought humility might reinforce gender stereotypes in a way that could disadvantage women. Table 3 shows the final list of 24 items defined as “essential” in describing professionalism. Figure 1 summarizes the item flow and decision logic over the three Delphi rounds.

#### Elements that generated discussion but did not reach consensus

Table 4 describes several non-consensus elements that generated multiple comments over the course of the study: these were related to physician wellness, social justice, access to care, and equity. Prioritizing patient over physician wellbeing drew criticism because it implied an unlimited prioritization of patient needs to the possible detriment of the physician’s wellbeing. Social justice was considered too broad in scope and potentially not directly applicable to physicians in different specialties or settings. Equal care and equity were supported but considered unrealistic in many healthcare settings.

**Table 4:** Selected panelist quotes about elements that did not reach consensus after survey rounds in the Professionalism Delphi Study, Shapiro Institute of Education and Research, 2023.

Element theme	Item wordings	Examples of panelist comments	Summary of identified issues
Physician wellness and wellbeing	Initial wording: Prioritizing patient over the physician’s own self-interests. Suggested rewordings: Prioritize patient care over one’s own needs. Practice self-care. Maintain physical and mental wellbeing in order to care for your patients.	“I think we need a new way to express this level of commitment without the physician being sacrificed.” “We should not be doing this chronically because of structural deficiencies in the system (i.e., hospital/office taking advantage of physician).”	While there was agreement that patient care was crucial, and sometimes called for personal sacrifices, panelists expressed the need for physicians to attend to their own wellbeing as well.
Social Justice	Initial wording: Promote social justice.	“Promote social justice is too broad. This can encompass working to improve healthcare system, communities, laws & policies, as well as working to ensure justice for individual patients/learners.” “I do not think all doctors need to be focused on maximizing access or promote social justice. What they cannot be is in the way of those things.”	Some participants felt this element was too broad in scope and potentially not directly applicable to physicians in different specialties or settings.
Access to care or equity	Initial wording: Manage limited resources for optimal patient outcomes. Suggested rewording: Maximize the quality of care even when access to needed resources.	“Commit to practice that provides equitable (not equal) care to all segments of the population. I think if we add this one, then “promote social justice” “maximize access to care” and “manage limited resources for optimal patient outcomes” can all be combined into one.” “Redundant.”	Participants felt the items about access to care, equity, and even social justice could all be grouped under equity or access to care. Managing limited resources was later replaced by maximizing access to care.

## Discussion

**Figure 2:**
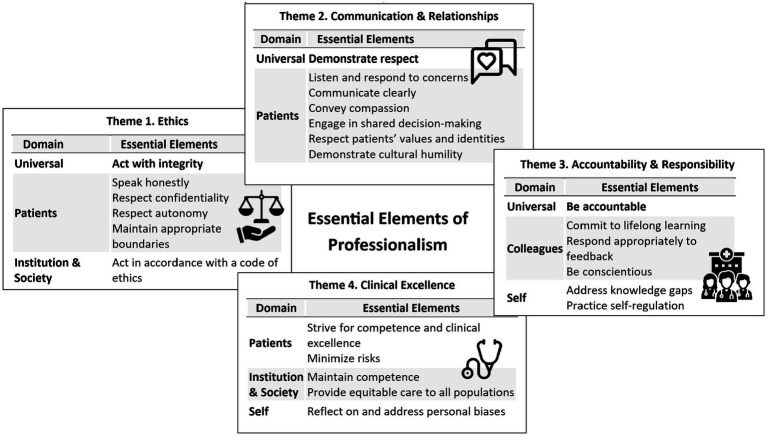
Professionalism Delphi results as defined by clinician leaders/educators (*n* = 39). Essential elements of professionalism, sorted by “commitments” (to patients, colleagues, institution and society, and self) and theme (ethics; communication and relationships; accountability and professional responsibility; and clinical excellence). Graphics from flaticon.com, designed by zero_wing, IwitoStudio, Awicon, Freepik from Flaticon.

The results of this modified Delphi study offer a consensus construct of professionalism in medicine for educators, one that accounts for contemporary social and cultural issues and the changing perspectives and needs of today’s generation of students and residents. Drawing on the perspectives of a diverse national panel of 39 experts from undergraduate and graduate medical education across eight medical schools in the U.S., we identified 24 “essential” elements of professionalism from an initial list of 51, the majority of which centered on commitments to patients—such as honesty, effective communication, compassion, respect for autonomy and confidentiality, and appropriate boundaries. The final elements were categorized into four domains—commitments to patients, colleagues, institutions, and society, self-highlighting the relational scope of professionalism across individual, interpersonal, and systemic contexts. Figure 2 presents a conceptual model summarizing these 24 elements across the four domains. For usability, we further organized the items into thematic groups (ethics, communication and relationships, accountability and professional responsibility, and clinical excellence).

Our combined quantitative and qualitative approach provided insights into the complexities and tensions in defining and applying tenets of professionalism, particularly as they relate to current generational, social, and cultural dynamics. The extended discussions during our 3-day, in-person conference highlighted how consensus definitions of professionalism raise the potential for both harms and benefits, how professionalism is inherently context-dependent, and how explicitly weaving positive examples of professionalism in teaching and assessment activities is needed to enhance learners’ development. Thus, the professionalism construct described here is not a “final” list of values and behaviors, but a dynamic, context-sensitive framework that guides teaching, assessment, and ongoing reflection in both classroom and clinical settings, particularly in the setting of U.S. medical schools in the current practice environment.

In discussing the *tensions* related to defining professionalism, participants expressed concerns, such as potential for harm to learners or learners’ reputations if definitions of professionalism were dichotomized as professional/unprofessional, used punitively or rigidly, taught in a purely didactic way, or imposed as rules to be followed. These findings align with recent studies and commentaries that highlight the potential for negative impacts of overly simplistic or biased conceptualizations of professionalism, particularly among learners from historically underrepresented groups in medicine (31–33). At the same time, participants expressed enthusiasm for having a shared understanding of core elements to guide teaching and feedback, and to serve as a springboard for discussion, reflection, and application in and to a variety of contexts—sentiments that are supported by research with learners and faculty in a variety of settings (12, 23).

Emphasis on explicit attention to *context* and *rationale* in teaching and assessing professionalism was also a recurrent theme. For example, participants emphasized that, while the final 24 consensus elements provided an initial framework for “what” to teach, each of these elements might manifest and be interpreted differently depending on the specific context. Ongoing dialog and reflection are required for learners to explore varied real-life or case-based scenarios and to achieve a deeper understanding of why and how different values and behaviors might, or might not, be perceived as professional. The same was true in discussions of the assessment of professionalism.

Participants shared common complaints from students who felt that professionalism was most likely to be highlighted/discussed in medical education when behavior was perceived as unprofessional, and this was often without consideration of the context in which the behavior or activity had occurred. (40), in an early review, noted that traditional approaches to teaching professionalism focused on more abstract definitions and often failed to account for the context-dependent nature of professional behaviors. In a more recent umbrella review (review of eight systematic reviews of professionalism interventions), (43) note considerable heterogeneity across interventions; reflection and role modeling were identified as common approaches, but there remains little mention of how context is explicitly incorporated into existing curricula.

Participants also enthusiastically advocated for a deliberate, ongoing emphasis on the *positive* manifestations and effects of professional behaviors, for example, pointing out and praising observable professional attitudes and behaviors among other clinicians, providing real-time feedback to compliment a learner on instances of their own professional behavior, or noting positive effects of professionalism, such as how it can engender trust and respect with patients and among team members. Some efforts to highlight this aspect of professionalism have been noted (e.g., 39), but additional guidance, curricular models, and evaluation of such models are needed.

In the survey, some elements, particularly those reflecting current debates, underwent multiple revisions, rewording, or did not reach consensus as essential for professionalism, possibly reflecting the ongoing tension between faculty and learners from different generations. For example, the concept of “cultural humility” was adopted after revision from “cultural proficiency,” aligning with evolving understandings that emphasize appreciation of differences, self-reflection related to one’s own biases, and curiosity rather than some abstract, unrealistic “mastery” of cultural issues (42), (41).

Similarly, the statement “prioritize patient care over the physician’s own self-interests”—itself a modification of “…physician’s own needs”—did not reach consensus as either essential or important to the construct of professionalism. Our discussants expressed strong opinions that this element, while capturing a long-held value of altruism as a core value of medical practice, was rightly challenged in the face of the COVID-19 pandemic (34) and could also increase the risk for burnout (35). This finding diverges from consensus statements from some national organizations’ endorsement of the “primacy of patient care” as a core principle that “…demands placing the interests of patients above those of the physician” (20) and “provid[ing] for the patient’s needs ahead of their own” (23), p. e1041. Thus, although altruism was acknowledged by participants as a key value, it was not included under the umbrella of “professionalism.” As one participant stated, “we need a new way to express this level of commitment without the physician being sacrificed.”

Equity and social justice, likewise, prompted animated discussion. While our panel enthusiastically supported equitable care and self-reflection on personal biases as essential features of professionalism, broader or more abstract commitments—such as advocacy for social justice—were viewed as less universally applicable across all specialties and settings. In this case, as in discussions of other elements, there was wide agreement that elements must be neither so abstract as to lack practical meaning, nor so specific as to be inflexible or not adaptable to most settings.

Finally, the element “dress appropriately” was ultimately deemed “important” rather than “essential,” given its dependence on local norms, the risk of subjective interpretation, and concern for possible bias or stereotyping, especially regarding diversity and inclusion. Participants agreed that a focus on foundational attitudes and behaviors—competence, compassion, ethics—offered a more meaningful and equitable basis for evaluating professionalism than prescriptive attention to attire.

When viewed in relation to international frameworks such as CanMEDS (36), the U.K. General Medical Council’s GOOD MEDICAL PRACTICE (37), and the World Federation for Medical Education (WFME) global standards (38), most of the 24 consensus elements align with universally recognized professional values—ethical practice, respect, communication, competence, and accountability. Where this framework may be most distinctively U.S.-specific is in its emphasis on equity, cultural humility, and contextual sensitivity, reflecting ongoing societal discourse and educational priorities within American medicine. The inclusion of physician wellbeing and boundaries as components of professionalism also mirrors recent attention within U.S. graduate medical education to sustainability and system-level support for professional identity formation. Future research should address whether and how this framework applies more broadly in other international or culturally diverse contexts.

Our findings must be considered in the context of several limitations. Our panelists were “on the ground” educators from American academic centers engaged daily in the support and development of medical students and residents; while we view this as a strength with respect to the expertise of the group, it did not represent a wider swath of administrators, interprofessional health educators, or patients and families. Our goal was to gain the perspective of educators in constant contact with learners to provide the best picture of the changes in the concept of professionalism resulting from rapid changes in our society. Although input from two students from two different schools was included in the qualitative data from the in-person discussions, they represented a minority of the larger Delphi panel. Future studies should triangulate educator and trainee viewpoints through parallel Delphi or mixed methods approaches to explore whether and why these groups hold similar or different conceptions of elements that are essential to professionalism.

## Conclusion

Building upon prior consensus statements from a broad range of medical professional organizations, as well as systematic and scoping reviews and studies in medical education in the US and internationally, our findings provide a consensus view generated by and for educators and education leaders from across the US. This consensus examination of professionalism emphasizes more general values of professionalism as well as the fluidity and context-dependence of the construct. Our process underlined the dynamic nature of our consensus construct of “professionalism,” portraying it not as a static set of guidelines but as a “living document” that can serve as a framework for ongoing dialog, reflection, and practical application by both learners and faculty in the diverse and real-world classroom and clinical settings.

## Data Availability

The original contributions presented in the study are included in the article/Supplementary material, further inquiries can be directed to the corresponding author.
