# Supplementary material for: Re-defining professionalism in medicine in an era of rapid change: a modified Delphi study
Source: Front Med (Lausanne). 2026 Jan 20;12:1686745. doi: 10.3389/fmed.2025.1686745 (PMC12864426; doi:10.3389/fmed.2025.1686745)
Supplement: Supplementary file 1 [file Image_1.pdf]

Supplemental Digital Content Appendix 1: **Search terms and primary references used to generate professionalism elements in Professionalism Delphi surveys.**

a. **Keyword and MeSH Search terms:**

(Professionalism[MeSH Terms] OR "professional conduct" OR "professional ethics" OR "professional identity" OR "professional standards" OR "professional behavior") AND ("2013/01/01"[Date - Publication] : "2023/12/31"[Date - Publication]) AND (Ethics, Medical[MeSH Terms] OR "ethical guidelines in medicine" OR "medical professional development" OR "consensus on professionalism") OR (education, medical, undergraduate [MeSH Terms]) or (education, medical, graduate [MeSH Terms])

b. **Primary references used to create Delphi surveys:**

Reference	Element Item*
Abdel-Razig S, Ibrahim H, Alameri H, Hamdy H, Haleeqa KA, Qayed KI, Obaid LO, Al Fahim M, Ezimokhai M, Sulaiman ND, Fares S, Al Darei MM, Shahin NQ, Al Shamsi NA, Alnooryani RA, Al Falahi SZ. Creating a Framework for Medical	**P12:** Attend to patient's family needs.<br>**IS2:** Maintain competence

Professionalism: An Initial Consensus Statement From an Arab Nation. J Grad Med Educ. 2016 May;8(2):165-72. doi: 10.4300/JGME-D-1500310.1. PMID: 27168882; PMCID: PMC4857522.

ABIM Foundation; ACP-ASIM Foundation; European Federation of Internal Medicine. Medical professionalism in the new millennium:

Berger AS, Niedra E, Brooks SG, Ahmed WS, Ginsburg S. Teaching Professionalism

Birden H, Glass N, Wilson I, Harrison M, **C9:** Be a role model for trainees. Usherwood T, Nass D. Teaching professionalism in medical education: a Best Evidence Medical Education (BEME) systematic review. BEME Guide No. 25. Med Teach. 2013 Jul;35(7):e125266. doi: 10.3109/0142159X.2013.789132. PMID: 23829342.

a physician charter. Ann Intern Med. 2002 Feb 5;136(3):243-6. doi: 10.7326/0003-4819-136-3200202050-00012. PMID: 11827500.

IS3: Commit to practice that provides equitable care to all segments of the population. **IS4:** Maximize access to care. **IS5:** Optimize the quality of care even when access to needed clinical resources is constrained.
IS6: Promote social justice.

in Postgraduate Medical Education: A Systematic Review. Acad Med. 2020 Jun;95(6):938-946. doi: 10.1097/ACM.0000000000002987. PMID: 31517687.

C11: Provide support to colleagues' intellectual, emotional, and physical well-being.
C15: Be responsive to colleagues' needs.

cultures: A narrative overview, Medical Teacher, 39:sup1, S8-S14, DOI: 10.1080/0142159X.2016.1254740

A. Al-Rumayyan, W. N. K. A. Van Mook, M. E. Magzoub, M. M. Al-Eraky, M. Ferwana, M. A. Khan & D. Dolmans (2017) Medical professionalism frameworks across non-Western

U1: Act with integrity.
U2: Demonstrate respect.

Cruess SR, Cruess RL. Professionalism and medicine's social contract. Focus on Health Professional Education: A Multi-Professional Journal. 2014;16(1):4-19. doi:https://doi.org/10.11157/fohpe.v16i1.52	**P8:** Convey compassion to patients.<br>**IS11:** Be transparent.
DeJong SM. Professionalism and Technology: Competencies Across the Tele-Behavioral Health and E-Behavioral Health Spectrum. Academic Psychiatry. 2018;42(6):800-807. doi:https://doi.org/10.1007/s40596-018-0947-x	**P13:** Using technology, including social media, appropriately.
DeLoughery EP. Professionalism Framings Across Medical Schools. J Gen Intern Med. 2018 May;33(5):610-611. doi: 10.1007/s11606-0184314-0. PMID: 29435728; PMCID: PMC5910349.	**C5:** Be punctual.
Birden H, Glass N, Wilson I, Harrison M, Usherwood T, Nass D. Defining professionalism in medical education: a systematic review. Med Teach. 2014 Jan;36(1):47-61. doi: 10.3109/0142159X.2014.850154. Epub 2013 Nov 19. PMID: 24252073.	**S3:** Practice self-reflection and commit to addressing your own biases.<br>**U3:** Be accountable.<br>Ellawala, A. T., Chandratilake, M., & de Silva, N. (2021). Using a consensus approach to develop a medical professionalism framework for the Sri Lankan context. Asia Pacific Scholar, 6(1).
	**P14:** Demonstrate respect for the values and identities of patients. Be
Cooke M, Irby DM, O'brien BC, Carnegie Foundation For The Advancement Of Teaching. Educating Physicians : A Call for Reform of Medical School and Residency. Jossey-Bass; 2010.	**IS10:** Commit to training and teaching the next generation.
**P10:** Engage in mutual decisionmaking.<br>**P17:** Prioritize patient care over physician's own self-interests. **IS1:** Act in accordance with a code of ethics.<br>**IS7:** Be compliant with regulatory standards.<br>**S2:** Practice self-regulation.	curious and engaged with patients.<br>**C2:** Respond to feedback appropriately.<br>**U4:** Act with humility.

Executive Committee; Benjamin IJ, Valentine CM, Oetgen WJ, Sheehan KA; Task Force 1; Brindis RG, Roach WH Jr, Harrington RA, Levine GN, Redberg RF, Broccolo BM, Hernandez AF; Task Force 2; Douglas PS, Piña IL, Benjamin EJ, Coylewright MJ, Saucedo JF, Ferdinand KC, Hayes SN, Poppas A; Task Force 3; Furie KL, Mehta LS, Erwin JP 3rd, Mieres JH, Murphy DJ Jr, Weissman G, West CP; Task Force 4; Lawrence WE Jr, Masoudi FA, Jones CP, Matlock DD, Miller JE, Spertus JA, Todman L; Task Force 5; Biga C, Chazal RA, Creager MA, Fry ET, Mack MJ, Yancy CW, Anderson RE. 2020 American Heart Association and American College of Cardiology Consensus Conference on Professionalism and Ethics: A Consensus Conference Report. J Am Coll Cardiol. 2021 Jun 22;77(24):3079-3133. doi: 10.1016/j.jacc.2021.04.004. Epub 2021 May 11. PMID: 33994057; PMCID: PMC8091280.	**P1:** Speak honestly with patients. **P2:** Demonstrate respect for patient confidentiality. **P3:** Strive for competency and clinical excellence. **P6:** Demonstrate respect for patient autonomy. **P9:** Establish appropriate boundaries in relationships with patients. **C6:** Commit to staying current in scientific knowledge. **IS9:** Commit to ongoing quality improvement.
Fong W, Kwan YH, Yoon S, Phang JK, Thumboo J, Leung YY, Ng SC. Assessment of medical professionalism using the Professionalism Mini Evaluation Exercise (PMEX) in a multi-ethnic society: a Delphi study. BMC Med Educ. 2020 Jul 14;20(1):225. doi: 10.1186/s12909-020-02147-9. PMID: 32664983; PMCID: PMC7362541.	**C12:** Use appropriate language. **S1:** Address gaps in knowledge.
Goddard VCT, Brockbank S. Re-opening Pandora's box: Who owns professionalism and is time for a 21st century definition? Med Educ. 2023 Jan;57(1):66-75. doi: 10.1111/medu.14862. Epub 2022 Jul 3. PMID: 35761477; PMCID: PMC10083973.	**C1:** Commit to lifelong learning. **C3:** Be conscientious. it

Hoonpongsimanont W, Sahota PK, Chen Y, Patel M, Tarapan T, Bengiamin D, Sutham K, Imsuwan I, Dadeh AA, Nakornchai T, Narajeenron K. Physician professionalism: definition from a generation perspective. Int J Med Educ. 2018 Sep 28;9:246-252. doi: 10.5116/ijme.5ba0.a584. PMID: 30269110; PMCID: PMC6387766.	**P4:** Listen and respond to patients' concerns.<br>**P7:** Communicate clearly to patients.<br>**P15:** Manage conflicts of interests.<br>**C7:** Demonstrate respect for other specialties.<br>**C8:** Demonstrate respect for other clinical professions.<br>**C16:** Practice situational awareness.
Jahan F, Siddiqui MA, Al Zadjali NM, Qasim R. Recognition of Core Elements of Medical Professionalism among Medical Students and Faculty Members. Oman Med J. 2016 May;31(3):196-204. doi: 10.5001/omj.2016.38. PMID: 27162590; PMCID: PMC4852086.	**P5:** Minimize risks to patients.
Koh, E.Y.H., Koh, K.K., Renganathan, Y. et al. Role modelling in professional identity formation: a systematic scoping review. BMC Med Educ 23, 194 (2023). https://doi.org/10.1186/s12909-023-04144-0	**S4:** Seek mentorship.
Lesser CS, Lucey CR, Egener B, Braddock CH 3rd, Linas SL, Levinson W. A behavioral and systems view of professionalism. JAMA. 2010 Dec 22;304(24):2732-7. doi: 10.1001/jama.2010.1864. PMID: 21177508.	**P11:** Demonstrate cultural humility.<br>**C10:** Address evidence of unprofessional behavior.<br>**IS8:** Debrief about error.
NEJM Knowledge+ Team. ACGME Core Competencies: Professionalism and Quality Care. NEJM Knowledge+. NEJM Knowledge+. Published January 12, 2017. https://knowledgeplus.nejm.org/blog/acgme-corecompetencies-professionalism/	**C13:** Request appropriate supervision.<br>**U5:** Demonstrate curiosity.

Helen O'Sullivan, Walther van Mook, Ray Fewtrell & Val Wass (2012) Integrating professionalism into the curriculum: AMEE Guide No. 61, Medical Teacher, 34:2, e64-e77, DOI: 10.3109/0142159X.2012.655610	**S6:** Cultivate emotional intelligence.
Puschel K, Repetto P, Bernales M, Barros J, Perez I, Snell L. "In our own words": Defining medical professionalism from a Latin American perspective. Educ Health (Abingdon). 2017 JanApr;30(1):11-18. doi: 10.4103/efh.EfH_4_16. PMID: 28707631.	**C14:** Be humble.
Sow CF, Mn C, Vd N. Developing a professionalism education framework at the institutional level with multidisciplinary consensus. Educ Med J. 2021;13(2):25-40.	doi:10.21315/eimj2021.13.2.3<br>**C4:** Dress appropriately. **P16:** Be timely in completing medical records.
Discussions from research team and suggestions provided by panelists in surveys.	**P18:** Demonstrate tolerance for ambiguity and uncertainty.<br>**S5:** Maintain physical and mental wellbeing in order to care for your patients.

*All identified articles cover a large range of elements, and we found that many elements overlapped across different articles. For conciseness, we highlight only each element once.

c. Example of tracking sheet for items:

This screenshot shows an example of our process of maintaining a list of all elements related to professionalism identified in our review of literature.

Source	Year	Type	Elements	Notes
2020 American Heart Association and American College of Cardiology Consensus Conference on Professionalism and Ethics A Consensus Conference Report	2020	Consensus statement	**Principles of Professionalism**: Primacy of patient welfare; Patient autonomy; Social justice. **Commitments**: Professional competence; Honesty with patients; Patient confidentiality; Maintaining appropriate relations with patients; Improving quality of care; Improving access to care; A just distribution of limited finite resources; Scientific knowledge; Maintaining trust by managing COIs; Professional responsibilities.	
Teaching Professionalism in Postgraduate Medical Education: A Systematic Review Arielle S. Berger, MD, Elizabeth Niedra, MD, Stephanie G. Brooks, Waleed S. Ahmed, MD, and Shiphra Ginsburg, MD, PhD	2020	Systematic review/GME	**Demonstrate commitment to patients**: Appropriate professional values and behavior; Commitment to excellence; Ethics in practice; Conflict of interest; Technology. **Demonstrate commitment to society:** Accountability; Patient safety/quality improvement. **Demonstrate commitment to the profession**: Adhere to professional/ethical codes; Respond to unprofessional behavior; Participate in peer assessment. **Demonstrate commitment to physician health and well-being**: Self-awareness and personal well-being; Manage personal and professional demands; Support colleagues in need	
ABIM Foundation, ACP Foundation and the European Federation of Internal Medicine: Medical professionalism in the new millennium: A physician charter	2005	Consensus statement	**Fundamental Principles** Principle of primacy of patient welfare. Principle of patient autonomy. Principle of social justice. **Professional Responsibilities** Commitment to professional competence; Commitment to honesty with patients; Commitment to patient confidentiality; Commitment to maintaining appropriate relations with patients.	
Swick, H. M. (2000). Toward a Normative Definition of Medical Professionalism. *Academic Medicine, 75* (6), 612-616.	2000	Perspective	**Behaviors**<br>Subordinate own interests to those of others; Adherence to high ethical and moral standards; Respond to societal needs; Physicians evince core humanistic values, including honesty andintegrity, caring and compassion, altruism and empathy, respect for others, and trustworthiness; Physicians exercise accountability for themselves and for their colleagues; Physicians demonstrate a continuing commitment to excellence; Physicians exhibit a commitment to scholarship and to advancing their field; Physicians deal [illegible]	
