# Supplementary material for: Re-defining professionalism in medicine in an era of rapid change: a modified Delphi study
Source: Front Med (Lausanne). 2026 Jan 20;12:1686745. doi: 10.3389/fmed.2025.1686745 (PMC12864426; doi:10.3389/fmed.2025.1686745)
Supplement: Supplementary file 2 [file Image_2.pdf]

Supplemental Digital Appendix 2: Examples of Professionalism Delphi Survey Round 1 & data report given to panelists

CENTER FOR EDUCATION
Shapiro Institute
BETH ISRAEL DEACONESS MEDICAL CENTER
HARVARD MEDICAL SCHOOL

Expert Consensus Study to Define Professionalism in Medicine

Many definitions of professionalism have been proposed in the medical literature. Our goal in this Delphi (consensus) study is to identify "essential" dimensions of professionalism that medical schools and hospitals can use to guide learners and faculty in developing professionalism among physicians, creating environments that support professionalism, and developing effective remediation strategies. Please note that this survey addresses only individual physician-level descriptions; other dimensions such as contextual, social, and environmental elements will be discussed at the conference.

In their book Educating Physicians: A Call for Reform of Medical School and Residency, Cooke, Irby, and O'Brien (2010) provide an overarching vision for the outcomes of medical education: "The physician we envision has, first and foremost, a deep sense of commitment and responsibility to patients, colleagues, institutions, society and self and an unfailing aspiration to perform better and achieve more." [page 41]

The forging of a professional identity, which incorporates the habits, thinking, and mindset of a physician, must be built upon a foundation of professionalism. Consequently, we believe that we must come to a common understanding of the essential elements of professionalism in medicine as the first step toward our goals of the Millennium Conference 2023.

This survey lists over 50 characteristics, behaviors or competencies, and values that have been included in definitions of professionalism, which we have culled from an extensive review of recent papers on this topic. We ask you to consider each item and rate whether it is an "essential"

element, "important" but not essential, or "not important" element to define professionalism. Topics that are "important" but not essential or "not important" may be important qualities or practices for clinicians, but do not fall under the definition of professionalism. We have organized the elements of professionalism into four categories:

1. Commitments to patients
2. Commitments to colleagues
3. Commitments to institution and society
4. Commitments to self

Finally, if you feel there are essential elements that are missing in this survey or that changes should be made in the wording of any of the elements, you will have the opportunity at the end of the survey to write this in or to provide any other feedback you think is important. Thank you!

Richard M. Schwartzstein, MD on behalf of the Shapiro Institute for Education and Research and the Millennium.

Expert Consensus Study to Define Professionalism in Medicine

Thank you for agreeing to participate! We expect it will take about 15 minutes to complete this survey.

2023 working group. Questions? Contact Amy Sullivan, Director for Education Research, at asulliv5@bidmc.harvard.edu.

**Commitments to Patients**	Essential	Important	Not Important
Treat patients in various situations with limited resources.	☐	☐	☐
Attend to patient's family needs.	☐	☐	☐
Convey compassion to patient.	☐	☐	☐
Engage in mutual decision-making.	☐	☐	☐
Prioritize patient care over one's own needs.	☐	☐	☐

Minimize risks to patients.	☐	☐	☐
Practice ongoing quality improvement.	☐	☐	☐
Manage conflicts of interest.	☐	☐	☐
Demonstrate respect for patient confidentiality.	☐	☐	☐
Establish appropriate boundaries in relationships with patients.	☐	☐	☐
Demonstrate respect for patient autonomy.	☐	☐	☐
Speak honestly with patients.	☐	☐	☐
Communicate clearly to patients.	☐	☐	☐
Listen and respond to patients' concerns.	☐	☐	☐
Use technology, including social media, appropriately.	☐	☐	☐
Demonstrate cultural proficiency.	☐	☐	☐
Be timely in completing medical records.	☐	☐	☐
Strive for competency and clinical excellence.	☐	☐	☐

Do you have any comments or suggestions for wording in the items listed above, including combining items? *(Please write in)*:

Are there essential attitudes, values, competencies, or behaviors missing in this category? *(Please write in)*:

**Commitments to Colleagues**	Essential	Important	Not Important
Practice situational awareness.	☐	☐	☐
Be responsive to colleagues' needs.	☐	☐	☐
Dress appropriately.	☐	☐	☐
Request appropriate supervision.	☐	☐	☐
Act with integrity.	☐	☐	☐
Use appropriate language.	☐	☐	☐
Address evidence of unprofessional behavior.	☐	☐	☐
Be humble.	☐	☐	☐
Commit to staying current in scientific knowledge.	☐	☐	☐
Respond to feedback appropriately.	☐	☐	☐
Be a role model for trainees.	☐	☐	☐
Commit to lifelong learning.	☐	☐	☐
Be conscientious.	☐	☐	☐
Demonstrate respect for other clinical professions.	☐	☐	☐
Be punctual.	☐	☐	☐
Demonstrate respect for other specialties.	☐	☐	☐
Offer help to colleagues who may be struggling.	☐	☐	☐

Do you have any comments or suggestions for wording in the items listed above, including combining items? *(Please write in)*:

Are there essential attitudes, values, competencies, or behaviors missing in this category? *(Please write in)*:

**Commitments to Institution & Society**	Essential	Important	Not Important
Act in accordance with a code of ethics.	☐	☐	☐
Promote social justice.	☐	☐	☐
Manage limited resources for optimal patient outcomes.	☐	☐	☐
Be accountable.	☐	☐	☐
Be transparent.	☐	☐	☐
Debrief about error.	☐	☐	☐
Maximize access to care.	☐	☐	☐
Maintain competence.	☐	☐	☐
Be compliant with regulatory standards.	☐	☐	☐

Do you have any comments or suggestions for wording in the items listed above, including combining items? *(Please write in)*:

Are there essential attitudes, values, competencies, or behaviors missing in this category? *(Please write in)*:

**Commitments to Self**	Essential	Important	Not Important
Practice self-care.	☐	☐	☐
Practice self-regulation.	☐	☐	☐

Address gaps in knowledge.	☐	☐	☐
Seek mentorship.	☐	☐	☐
Be accountable.	☐	☐	☐
Cultivate emotional intelligence.	☐	☐	☐
Practice self-reflection.	☐	☐	☐

Do you have any comments or suggestions for wording in the items listed above, including combining items? *(Please write in)*:

Are there essential attitudes, values, competencies, or behaviors missing in this category? *(Please write in)*:

In this last item, please take the opportunity to share any comments or suggestions that you have about how you would like to define professionalism. Thank you for completing this survey!

MC2023 Delphi Study: Round 2 Data Report Summary

This data report summarizes results from 34 completed surveys and 2 incomplete survey responses. Datasets were collected online from April 7-26, 2023.

Items that reached ≥70% consensus for *essential* after survey rounds 1&2

Commitments to Patient:

- Speak honestly with patients.
- Demonstrate respect for patient confidentiality.
- Strive for competency and clinical excellence.
- Listen and respond to patients' concerns.
- Minimize risks to patients.
- Demonstrate respect for patient autonomy.
- Communicate clearly to patients.

- Demonstrate cultural humility.
- Convey compassion to patient.
- Establish appropriate boundaries in relationships with patients.
- Engage in mutual decision-making.

Commitments to Colleagues:

- Act with integrity.
- Commit to lifelong learning.
- Respond to feedback appropriately.
- Be accountable to the team.
- Be conscientious.

Commitments to Institution & Society:

- Be accountable.
- Act in accordance with a code of ethics.
- Maintain competence.
- Optimize the quality of care even when access to needed clinical resources is constrained.

Commitments to Self:

- Address gaps in knowledge.
- Practice self-regulation.
- Practice self-reflection, query your own biases, and be open to addressing them.

Items that reached ≥70% consensus for *important* after survey rounds 1&2

- Attend to patient's family needs.
- Using technology, including social media, appropriately.
- Dress appropriately.
- Maximize access to care.
- Seek mentorship.

Commitments to Patients: Open Response Summary

Comments on item rewording: Frequently mentioned elements

Item 6: Demonstrate an attitude of curiosity and engagement with patients (4 comments)

Respondents interpreted this item differently with both positive and negative connotations. One noted that it is a behavior that would be challenging to observe and assess:

- “First new suggested item is double barreled - curiosity and engagement are two separate things. I agree clinicians should be engaged, but it is not always appropriate to be curious.”

- “In terms of defining professional behavior, these "demonstrate attitude" and "demonstrate tolerance for ambiguity" are really hard to actually capture as behaviors. I think that "demonstrate engagement with patients" is reasonable, but curiosity, ambiguity and uncertainty are all internal activities and hard to translate into observable behaviors.”

- “I really like the new item about curiosity and engagement because of what I think is behind it - it is critical to engage patients where they are and respect them for who they are. The word respect comes up in the accepted items in relation to confidentiality and autonomy, but those items to not to me talk about respecting them as a person for their identity, perspective, and life experiences.”

- “Demonstrating attitudes is vague to me and my sense is it would look different in different cultural settings. Showing curiosity and engagement with patients is more aligned with how to build rapport than how to be professional.”

Item 2: Manage conflicts of interests (4 comments)

Respondents felt this item needed more description explaining what constitutes a ‘conflict of interest.’ Examples include:

- “I am not sure what ‘manage conflicts of interest’ means.”

- “Would change ‘manage conflicts of interest’ to ‘recognize and manage conflicts of interest.”

Overall, when respondents labeled an item as problematic, it is because they felt the item lacked context-specific details.

Suggested new element: Most frequently suggested new element entailed *incorporating patient values/perspectives into the professional practice* (3 responses):

- “It may be included in the above, but I do want to make sure we're including the idea of incorporating patient values/ perspective into their care.”

- “I feel the idea of respect for patients is missing.”

- “…2) The ability to listen and hear the values and identities of your patients…5) The ability to solicit and hear feedback, using it to drive personal improvement…”

Items that did not reach consensus after survey rounds 1&2

*No items reached consensus in the NOT IMPORTANT category in Survey Rounds 1 and 2.

**P15: Manage conflicts of interest.**	% of Agreement	Count
1 - Essential	50.0%	17
2 - Important but not essential	41.2%	14
3 – Not important	8.8%	3
**TOTAL**	100%	34

P17: Prioritize patient care over physicians' own self interests.	% of Agreement	Count
1 - Essential	47.1%	16
2 - Important but not essential	47.1%	16
3 – Not important	5.8%	2
**TOTAL**	100%	34

P16: Be timely in completing medical records.	% of Agreement	Count
1 - Essential	29.4%	10
2 - Important but not essential	67.6%	23
3 – Not important	3.0%	1
**TOTAL**	100%	34

P16: Be timely in completing medical records.	% of Agreement	Count
1 - Essential	29.4%	10
2 - Important but not essential	67.6%	23
3 – Not important	3.0%	1
**TOTAL**	100%	34

P14: Demonstrate an attitude of curiosity and engagement with patients.	% of Agreement	Count
1 - Essential	54.3%	19
2 - Important but not essential	37.2%	13
3 – Not important	8.5%	3
TOTAL	100%	35

P18: Demonstrate tolerance for ambiguity and uncertainty.	% of Agreement	Count
1 - Essential	48.6%	17
2 - Important but not essential	51.4%	18
3 – Not important	0%	0
TOTAL	100%	35
