# Supplementary material for: Re-defining professionalism in medicine in an era of rapid change: a modified Delphi study
Source: Front Med (Lausanne). 2026 Jan 20;12:1686745. doi: 10.3389/fmed.2025.1686745 (PMC12864426; doi:10.3389/fmed.2025.1686745)
Supplement: Supplementary file 3 [file Image_3.pdf]

Supplemental Digital Appendix 3: In-person meeting handout to facilitate consensus discussions & the final Delphi survey in Round 3

Commitments to Patients: Item Summary after Round 2

Reached ≥70% Consensus for Essential:

- Speak honestly with patients.
- Demonstrate respect for patient confidentiality.
- Strive for competency and clinical excellence.
- Listen and respond to patients' concerns.
- Minimize risks to patients.
- Demonstrate respect for patient autonomy.
- Communicate clearly to patients.
- Demonstrate cultural humility.
- Convey compassion to patients.
- Establish appropriate boundaries in relationships with patients.
- Engage in mutual decision-making.

Reached ≥70% Consensus for Important:

- Attend to patient's family needs.
- Using technology, including social media, appropriately.

* No item reached consensus at the Not Important rating.

Items that did not reach consensus

No Consensus Items	Essential	Important	Not Important
1. Manage conflicts of interests.	☐	☐	☐
2. Be timely in completing medical records.	☐	☐	☐
3. Prioritize patient care over physicians' own self-interests.	☐	☐	☐
4. Demonstrate an attitude of curiosity and engagement with patients.	☐	☐	☐
5. Demonstrate tolerance for ambiguity and uncertainty.	☐	☐	☐

Item rewording & new element suggestions from survey participants:

1. Manage conflicts of interests:
Some respondents thought this statement was vague. One suggested changing the statement to "Recognize and manage conflicts of interests."

4. Demonstrate an attitude of curiosity and engagement with patients:
Curiosity and engagement are different attributes; can agree with one but not the other.

New element: Incorporate patient values/perspectives into professional practice.

- "I feel the idea of respect for patients is missing."
- "…2) The ability to listen and hear the values and identities of your patients…"
- "It may be included in the above, but I do want to make sure we're including the idea of incorporating patient values/ perspective into their care."

1

Commitments to Colleagues: Item Summary after Round 2

Reached ≥70% Consensus for Essential:

- Act with integrity.
- Commit to lifelong learning.
- Respond to feedback appropriately.
- Be accountable to the team.
- Be conscientious.

Reached ≥70% Consensus for Important:

- Dress appropriately.

* No item reached consensus at the Not Important rating.

Items that did not reach consensus

No Consensus Items	Essential	Important	Not Important
6. Be punctual.	☐	☐	☐
7. Commit to staying current in scientific knowledge.	☐	☐	☐
8. Demonstrate respect for other specialties.	☐	☐	☐
9. Demonstrate respect for other clinical professions.	☐	☐	☐
10. Be a role model for trainees.	☐	☐	☐
11. Address evidence of unprofessional behavior appropriately.	☐	☐	☐
12. Practice situational awareness.	☐	☐	☐
13. Be humble.	☐	☐	☐
14. Provide support to colleagues' intellectual, emotional, and physical wellbeing.	☐	☐	☐

Item rewording & new element suggestions from survey participants:

- Combine #8 and #9: "…respect for other clinical professions *and* other specialties."
- 12. Practice situational awareness: Too ambiguous. Needs more context.
- What does **appropriately** mean?
  "How to define this independent of one's own biases and expectations?"

2

Commitments to Institution & Society : Item Summary after Round 2

Reached ≥70% Consensus for Essential:

- Be accountable.
- Act in accordance with a code of ethics.
- Maintain competence.
- Optimize the quality of care even when access to needed clinical resources is constrained.

Reached ≥70% Consensus for Important:

- Maximize access to care.

* No item reached consensus at the Not Important rating.

Items that did not reach consensus

No Consensus Items	Essential	Important	Not Important
15. Promote social justice.	☐	☐	☐
16. Be compliant with regulatory standards.	☐	☐	☐
17. Be transparent.	☐	☐	☐
18. Manage limited resources for optimal patient outcomes.	☐	☐	☐
19. Debrief about error.	☐	☐	☐
20. Commit to ongoing quality improvement.	☐	☐	☐
21. Commit to practice that provides equitable care to all segments of the population.	☐	☐	☐
22. Commit to training and teaching the next generation.	☐	☐	☐

Item rewording & new element suggestions from survey participants:

- Changed *equal* to **equitable care** in #23: 9 participants suggested this change.
- 15. Promote social justice: Too broad and vague. Needs more context.
- 19. Debrief about error: Too broad. Lacking details.

Commitments to Self: Item Summary after Round 2

Reached ≥70% Consensus for Essential:

- Address gaps in knowledge.
- Practice self-regulation.

Reached ≥70% Consensus for Important:

- Seek mentorship.

* No item reached consensus at the Not Important rating.

Items that did not reach consensus

No Consensus Items	Essential	Important	Not Important
23. Maintain physical and mental wellbeing in order to care for your patients.	☐	☐	☐
24. Cultivate emotional intelligence.	☐	☐	☐
25. Practice self-reflection, query your own biases, and be open to addressing them.	☐	☐	☐

Item rewording & new element suggestions from survey participants:

23. Maintain physical and mental wellbeing in order to care for your patients: This item was reworded from Round 1. However, some respondents still felt the wording is problematic. Examples:

- "Should rephrase "Maintain physical and mental well-being" to avoid implication that you must be healthy when not all of us have that luck. Perhaps "Prioritize personal physical and mental wellbeing..." or something like that that gets at optimization of whatever luck you are given."
- "Maintain physical and mental well-being in order to best care for your patients."
- "Maintain…in order to care for your patients" is a little vague. It sounds like the reason to take care of ourselves is so we can take care of patients. I don't think that's quite right."

Expert Consensus Study to Define Professionalism in Medicine Round 3

IMPORTANT!!! PLEASE TAKE A MOMENT TO READ
Thank you for participating in Round 3 of the Delphi study! For this **last survey**, we have a few final questions for you.

Here is what we ask you to consider in this last round:

1. Participants have suggested **rewording** for a few items that were close to, but did not reach, consensus. We present here the original items and 1-2 suggested rewordings for you to respond to. We will ask you to (a) select the wording you prefer, and then (b) rate that item "essential," "important but not essential," or "not important" for the definition of professionalism.

2. Now that we have had a few days together to discuss professionalism and professional identity formation, is there anything that you wish to comment on or any final thoughts you would like to share? We have an open-ended item for you to share your thoughts.

We will send you a full report later this month, at which time you will have another opportunity to share any additional comments you have on the results.

Thank you!

Richard M. Schwartzstein, MD on behalf of the Shapiro Institute for Education and Research and the Millennium 2023 working group

Questions? Contact Amy Sullivan, Director for Education Research, at asulliv5@bidmc.harvard.edu

1. A number of items were considered to cut across all categories (patient, colleagues, institution and society, and self). Please indicate the extent to which you agree or disagree that this is a "universal" element of professionalism. **If you say "agree," this means that you think it is an element that cuts across all categories. If you say "disagree," this indicates that you believe this is a category-specific item**.

	Strongly disagree it is universal	Somewhat disagree it is universal	Neither agree nor disagree	Somewhat agree it is universal	Strongly agree it is universal
**Act with integrity.**	☐	☐	☐	☐	☐
**Demonstrate respect.**	☐	☐	☐	☐	☐
**Act with humility.**	☐	☐	☐	☐	☐
**Demonstrate curiosity.**	☐	☐	☐	☐	☐

Do you have any comments you want to make about the items above, or anything else to add?

---

2. **Rewording**. The following items have suggestions for rewording. We first ask you to choose the version you prefer. We then ask you to rate the item as essential, important, or not important for professionalism.

Item 1. Please choose the wording you prefer:

- o **Original item:** Demonstrate an attitude of curiosity and engagement with patients.
- o **Alternative wording 1:** Demonstrate respect, curiosity, and engagement with patients.
- o **Alternative wording 2:** Demonstrate respect for values and identities of patients. Be curious and engaged with patients.
- o **Alternative wording 3:** Be curious and engaged with patients.

Given the wording you chose for Item 1, how would you rate this item?

- o Essential for professionalism
- o Important but not essential for professionalism
- o Not important for professionalism

Item 2. Please choose the wording you prefer:

- o **Original item:** Maintain physical and mental wellbeing in order to care for your patients.

- **Alternative wording 1:** Commit to wellbeing.
- **Alternative wording 2:** Prioritize personal, physical, and mental wellbeing in order to care for your patients.

Given the wording you chose for Item 2, how would you rate this item?

- Essential for professionalism
- Important but not essential for professionalism
- Not important for professionalism

Item 3. Please choose the wording you prefer:

- **Original item:** Practice self-reflection, query your own biases, and be open to addressing them.
- **Alternative wording:** Practice self-reflection and commit to addressing your own biases.

Given the wording you chose for Item 3, how would you rate this item?

- Essential for professionalism
- Important but not essential for professionalism
- Not important for professionalism

Item 4. This item was very close to consensus. Please provide a final rating of this item:

Commit to practice that provides equitable care to all segments of the population.

- Essential for professionalism
- Important but not essential for professionalism
- Not important for professionalism

Is there anything that you wish to comment or any final thoughts you would like to share?
